# Point-of-Care Ultrasound for the Diagnosis of Congestive Kidney Due to Severe Acidemia: A Case Report

**DOI:** 10.7759/cureus.57096

**Published:** 2024-03-27

**Authors:** Kana Shirai, Masahiko Yazawa, Keisuke Yoshida, Yugo Shibagaki

**Affiliations:** 1 Department of Medicine, St. Marianna University, Kawasaki, JPN

**Keywords:** severe acidemia, acute kidney injury, vexus, congestive kidney, point-of-care ultrasound

## Abstract

A 51-year-old woman with mitochondrial myopathy and congestive heart failure with reduced left ventricular ejection fraction was admitted due to loss of appetite and progressive frailty. She presented with acute kidney injury (AKI) and severe acidemia. Given her medical history and physical examination (jugular vein distention was not obvious), prerenal causes (hypovolemia/hypotension) of AKI were considered most likely. However, with a significantly elevated N-terminal pro-b-type natriuretic peptide level of 14,700 pg/mL, a congestive kidney was also considered. Bedside echocardiography showed no evidence of low output syndrome, whereas venous excess ultrasound (VExUS) score was assessed as Grade 2 (moderate congestion). In addition to administering fluids for the suspected prerenal causes (hypovolemia/hypotension), sodium bicarbonate was administered suspecting a negative impact of severe acidemia on cardiac function. With the improvement of acidemia and only a small volume of fluid therapy, there was a rapid improvement in AKI with the normalization of the VExUS score. This suggested that the main cause of AKI was congestive kidney. In this case, VExUS helped us make a correct diagnosis of acidemia-induced congestive kidney rather than hypovolemia as a cause of AKI, leading to the appropriate treatment.

## Introduction

Point-of-care ultrasonography (POCUS) is a novel approach where the treating physician directly performs ultrasonography at the bedside, enabling rapid understanding of the patient’s condition, diagnosis, and treatment [1]. POCUS can swiftly identify structural or functional abnormalities in internal organs, allowing for a more accurate assessment of congestion in organs, tissues, and blood vessels [2]. In patients with kidney disease, where the clinical assessment mostly relied on vital signs and laboratory findings, POCUS enables the visual evaluation of changes in organ blood flow due to hemodynamic shifts, enhancing the accuracy of diagnosis. For instance, one of the challenges in diagnosing hemodynamic-related acute kidney injury (AKI) is determining how to represent the increase in venous pressure (congestion). Of the currently available indicators, such as central venous pressure, cumulative fluid balance, changes in body weight, and physical findings of peripheral edema, each has its caveats [3,4]. Venous excess ultrasound (VExUS), one method in POCUS, was developed to estimate venous congestion by assessing the inferior vena cava (IVC), hepatic vein, portal vein, and renal vein to yield a VExUS grade [5]. Here, we present a case in which VExUS strongly suggested renal congestion, which was otherwise not suspected, leading to rapid and appropriate management of the case.

## Case presentation

A 51-year-old woman with a history of mitochondrial myopathy with heart failure with reduced ejection fraction was transferred to the emergency room for progressive frailty after several days of appetite loss of unknown cause. Diarrhea, fever, and vomiting were not observed. She was on the following medications: levetiracetam 250 mg bid, 98% taurine 1 g tid, mecobalamin 500 µg tid, lansoprazole 15 mg qd, pimobendan 1.25 mg qid, bisoprolol 0.625 mg qd, furosemide 10 mg qd, candesartan 2 mg qd, and spironolactone 50 mg qd.

**Figure 1:**
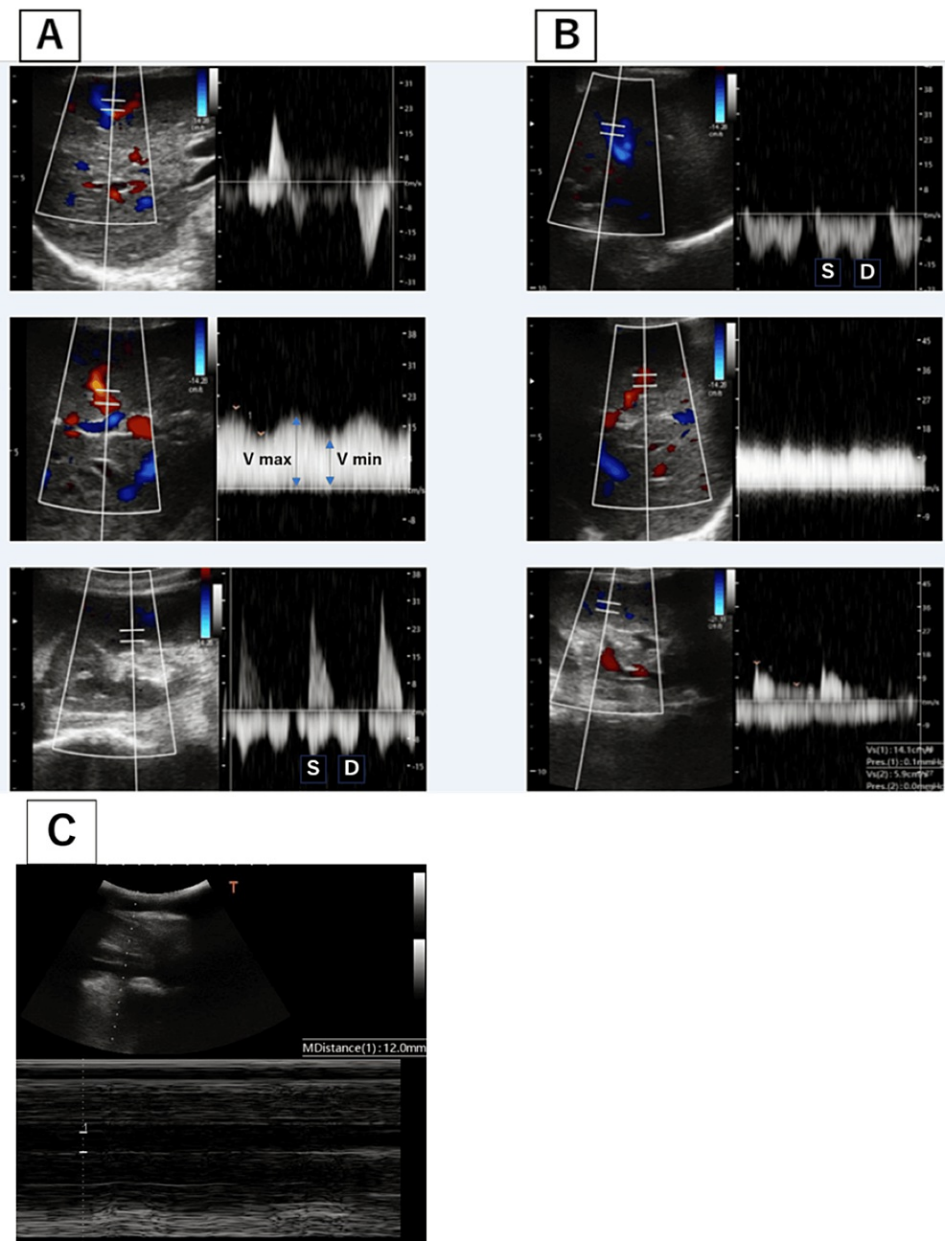
VExUS score before and after alkali administration. (A) Before alkali administration. (B) After alkali administration. (C) IVC. VExUS: Grade 2, mildly abnormal. VExUS: Grade 1, normal. VExUS = venous excess ultrasound; IVC = inferior vena cava

Upon arrival, she had a blood pressure of 79/49 mmHg, which was slightly lower than her baseline systolic blood pressure of 90 mmHg, a heart rate of 73 beats per minute, and oxygen saturation of 94% on room air. Physical examination revealed no crackles, lower leg edema, or cold extremities. Her serum creatinine level was 4.2 mg/dL (baseline of 1.0). There was no creatine phosphokinase elevation. No myalgia was noted, and there were no elevations in hepatobiliary enzymes. Urine output was scant at 50 mL over four hours, indicating oliguric AKI. Arterial blood gas showed a pH of 6.98, pCO_2_ of 33.3, HCO_3_ of 7.6, and an anion gap of 15.6, indicating severe metabolic acidosis. Lactate was 1.4 mmol/L (not elevated), there were no urine ketones, and the osmolar gap was 5.6 (not elevated). Acidosis was not originally observed. The patient had respiratory acidosis, with an unknown cause, as the patient was not impaired at the time of presentation, had no history of smoking, and had no abnormalities in the lung fields. Regarding AKI, the ultrasound did not show any post-renal findings, and there were no recent changes in medications. The urine sediment was bland with fractional excretion of sodium of 0.7% and fractional excretion of urea nitrogen of 5.5%, indicating a prerenal mechanism. Based on her medical history, prerenal causes due to hypovolemia from loss of appetite and/or hypotension due to hypovolemia seemed most likely at first. There was no increased inflammatory response and no obvious infection on imaging findings. Echocardiography showed no D-shape or pericardial effusion. However, there was a significant increase in N-terminal pro-b-type natriuretic peptide (NT-proBNP) to 14,700 pg/mL, which suggested congestive heart failure. Despite that, chest X-ray did not reveal any obvious congestion or pleural effusion, and there was no need for oxygen supplementation. A cardiologist performed a bedside cardiac ultrasound; however, unfortunately, no measurements were documented. To rule out the possibility of congestive kidney failure, a VExUS was performed. IVC was approximately 12 mm, which did not meet the criteria (>2 cm) to proceed to VExUS. However, we still proceeded with VExUS because of the patient’s small body size, which we often encounter in Japanese individuals with clinical congestion, lack of respiratory variability, and high level of NTproBNP, which suggested congestion. Hepatic Doppler was considered mildly abnormal as the systolic component was lower in magnitude than the diastolic component, but still toward the liver; it is considered severely abnormal when the systolic component is reversed (toward the heart). Portal Doppler was considered mildly abnormal as a variation in the velocities during the cardiac cycle of 30 to <50 % is observed; it is considered severely abnormal when a variation of ≥50 % is seen. Intrarenal venous Doppler was considered mildly abnormal as it is discontinuous with a systolic and diastolic phase; it is considered severely abnormal when it is discontinuous with only a diastolic phase seen during the cardiac cycle [5]. Although there was no EKG synchronization, and no abnormal findings in the hepatic veins, the findings of the portal and renal veins were assessed as grade 2 (mildly abnormal), suggesting the possibility of a congestive kidney (Figure 1). We suspected that acidemia was the cause of congestion (heart dysfunction), and thus 120 mL of 7% sodium bicarbonate was administered along with 260 mL of isotonic fluid. This resulted in an improvement in acidemia to a pH of 7.22, HCO_3_ of 19, and an increase in systolic blood pressure above the 90 mmHg range. One hour after administering the sodium bicarbonate, the urine output began to increase, reaching 450 mL in an hour even though no diuretics were administered. NT-proBNP was 14,700 pg/mL before alkalinization, decreased to 5,780 pg/mL after alkalinization followed by significantly increasing urine output, and then further decreased to 2,254 pg/mL by the following day. At this point, the IVC measured 0-4 mm with respiratory variation, and the VExUS grade was 1 (Figure 1). The fluid replacement was then discontinued, and by the next day, arterial blood gas analysis showed further improvement in acidosis with a pH of 7.2, pCO_2_ of 52 mmHg, and HCO_3_ of 23.3. Moreover, the creatinine level improved to 1.16, indicating recovery from AKI. Given that only a relatively small volume of fluid was administered, it was not hypovolemia, but the congestive kidney, caused by a relative decrease in cardiac function due to severe acidemia, was the main cause of AKI.

## Discussion

The diagnosis of hemodynamic-associated AKI is often challenging due to the lack of established methods to assess the hemodynamic state of the target organ. The so-called “pre-renal” AKI, which is caused by low perfusion in the renal vasculature, can occur not only in pre-renal states (such as hypovolemia or hypotension) but also in post-renal states, as well as in conditions such as abdominal compartment syndrome or congestive kidney, where renal venous/interstitial pressure is significantly elevated. This has led to the proposal of categorizing these conditions under the umbrella of “low-flow” AKI [6].

The diagnostic dilemma lies in the absence of reliable tests/procedures to accurately, timely, and easily evaluate the hemodynamic state of renal vasculature (kidney perfusion). Recently, POCUS has proven to be useful in this context. In particular, the VExUS technique established by Beaubien-Souligny et al. has been demonstrated to be effective in assessing the hemodynamic state of target organs, such as kidney congestion [6]. VExUS combines the measurements of IVC diameter and venous Doppler waveforms of the portal, hepatic, and interlobular renal veins to assess the degree of organ congestion. The presence of at least two severe alterations of the hepatic vein, portal vein, or intra-renal venous flow on pulse-wave Doppler ultrasound with an IVC ≥2 cm of diameter at intensive care unit admission after cardiac surgery indicates a high risk of postoperative AKI. Their report showed that the positive likelihood ratio for predicting AKI was higher with VExUS than with invasively measured central venous pressure. These findings have attracted significant interest among researchers in cardiology, critical care, and nephrology [5,7].

In this case, initially, prerenal AKI due to hypovolemia/hypotension was clinically suspected as the cause of the AKI. Although NT-proBNP was elevated and IVC was dilated, anemia, uremia, and secondary hyperparathyroidism associated with chronic kidney disease are known to adversely affect the heart and increase NT-proBNP, while IVC can be affected by cardiac function and/or IVC parietal compliance other than volume status and thus was not useful as an indicator of fluid volume [8,9]. The only differing factor was the elevated NT-proBNP, which led to the consideration of congestive kidney in the differential diagnosis. Although the bedside echocardiography was done before alkali administration, with eyeball diagnosis of cardiac dysfunction, unfortunately, without documented measurements, led us to proceed with VExUS. Performing VExUS led us to the diagnosis of congestion and facilitated the early treatment of acidemia as a cause of congestion. Indeed, the rapid improvement in AKI concurrent with the correction of acidemia, and the substantial urine output that did not align with hypovolemia, supported the diagnosis of congestive kidney.

The decrease in cardiac function due to acidemia is well-known, specifically, below pH 7.2, negative inotropic effects on the heart outweigh positive inotropic effects and may reduce cardiac function. For the vascular system, it constricts veins and increases venous perfusion to the heart. Arteries dilate, which may cause hypotension [10]. However, there is limited clinical evidence of the improvement with alkali administration [11]. Except for L-lactic acidosis due to tissue hypoxemia, alkali administration is considered unnecessary in most cases, and unnecessary alkali administration can cause adverse events such as hypokinemia and paradoxical intracellular acidosis. However, in this case, VEXUS showed significant congestion, which was the rationale for administering alkali, considering the negative inotropic effect of acidemia on the heart. Without the diagnosis provided by VExUS, there might have been an inclination to administer a greater volume of fluids or hesitation in administering alkali for acidemia, which remains a contentious issue. In implementing VExUS, it should be noted that diagnostic accuracy may decrease in patients with complicating factors such as increased intra-abdominal pressure [12]. However, this case did not present any of these complicating factors. Additionally, interpreting VExUS in isolation could potentially lead to erroneous clinical judgments [12]. Therefore, it is crucial to consider VExUS findings in conjunction with other clinical observations (physical examination and test results).

## Conclusions

This case report demonstrates the usefulness of POCUS in the diagnosis and management of hemodynamic-associated AKI. As the volume status of the patient was ambiguous (in this case, clinical course/history, physical examination was discordant with BNP value/IVC diameter), VExUS was useful to include/exclude the volume status (congestion of kidneys). This example emphasizes the importance of personalized, real-time imaging diagnostic tools in the management of AKI.
